# Can Yogic Breathing Techniques Like *Simha Kriya* and *Isha Kriya* Regulate COVID-19-Related Stress?

**DOI:** 10.3389/fpsyg.2021.635816

**Published:** 2021-04-15

**Authors:** Manjari Rain, Balachundhar Subramaniam, Pramod Avti, Pranay Mahajan, Akshay Anand

**Affiliations:** ^1^Department of Neurology, Post Graduate Institute of Medical Education and Research, Chandigarh, India; ^2^Center for Anesthesia Research Excellence, Beth Israel Deaconess Medical Center, Harvard Medical School, Boston, MA, United States; ^3^Department of Biophysics, Post Graduate Institute of Medical Education and Research, Chandigarh, India; ^4^Department of Hospital Administration, Post Graduate Institute of Medical Education and Research, Chandigarh, India; ^5^Centre for Mind Body Medicine, Post Graduate Institute of Medical Education and Research, Chandigarh, India; ^6^Centre of Phenomenology and Cognitive Sciences, Panjab University, Chandigarh, India

**Keywords:** COVID-19, *Isha Kriya*, novel coronavirus, *Simha Kriya*, yogic breathing, breathing techniques

## Abstract

The global impact of Coronavirus Disease 2019 (COVID-19) is tremendous on human life, not only affecting the physical and mental health of population but also impacting the economic system of countries and individual itself. The present situation demands prompt response toward COVID-19 by equipping the humans with strategies to overcome the infection and stress associated with it. These strategies must not only be limited to preventive and therapeutic measures, but also aim at improving immunity and mental health. This can be achieved by yogic breathing techniques. In this perspective, we emphasize the importance of yogic breathing, *Simha Kriya* and *Isha kriya*, the simple yet effective breathing techniques.

## Introduction

Traditional yogic systems that involve breathing exercises (*Pranayama*), yoga postures (*Asanas*), chants and meditation have been known to reduce physical and mental stress, enhance lung functions, and immunity (Hakked et al., 2017). Mindful meditation for a single day is able to decrease the gene expression of histone deacetylase genes (HDAC 2, 3, and 9) and pro-inflammatory genes (RIPK2 and COX2) in experienced meditators (Kaliman et al., 2014). Interestingly, Yoga practice has shown to decrease the oxidative stress markers and up-regulate the expression of telomerase genes, such as telomerase reverse transcriptase and telomerase RNA, suggesting increased cellular viability and reduced cellular aging (Duraimani et al., 2015). Studies have shown that Yoga therapy/meditation can improve health and well-being of a diseased individual by reducing stress besides boosting the immune response (Thirthalli et al., 2013; Danhauer et al., 2019; Tang et al., 2020; Venkatesh et al., 2020). Mindfulness-based cognitive therapy can significantly reduce stress, depression and anger, when administered in-person, or virtually (Schanche et al., 2020; Kuo et al., 2021; Orosa-Duarte et al., 2021; Strauss et al., 2021). Similar to Yoga, mindfulness-based techniques also have immune modulatory effects (Reich et al., 2017; Andrés-Rodríguez et al., 2019).

Some studies suggest the role of yogic controlled breathing in reducing the stress levels, controlling mood fluctuations such as anxiety and depression, and at the same time improving quality of life by motor co-ordination, cognitive performance, heart rate variability, and more (Bernardi et al., 2000; Sharma et al., 2014; Gonçalves et al., 2016; Erdoğan Yüce and Taşcı, 2020). Systolic and diastolic blood pressure is improved immediately after practicing *Pranayama* (Pramanik et al., 2009; Bhavanani et al., 2011). It is notable that practicing Yoga, especially breathing techniques, may be beneficial to control anxiety, stress and to some extent modulate immune response. However, validating such technique in a clinical set up is obligatory.

Enhanced anxiety and stress levels were reported worldwide in Coronavirus Disease 2019 (COVID-19) pandemic amongst health care workers (HCWs), quarantined individuals (suspected cases), convalescent individuals and COVID-19 positive cases due to sudden rise in cases and inability to manage COVID-19 with medication available in the early stages of pandemic (Luo et al., 2020; Ornell et al., 2020; Pfefferbaum and North, 2020; Halperin et al., 2021). Psychological stress has been reported to increase during the lockdowns, quarantine periods and after stay-at-home orders (Ozamiz-Etxebarria et al., 2020). Anxiety and stress have also increased among people due to recent vaccination drive; some are anxious while waiting for their first or second vaccine shot while others have doubts on efficacy of vaccines, which are still under trial. Psychological stress is a known factor which is detrimental to immune modulation and stress can diminish the immune response post vaccination (Glaser et al., 1998; Segerstrom and Miller, 2004; Pedersen et al., 2009; Madison et al., 2021). It is widely believed that stress associated with COVID-19 may worsen the severity of infection and may render otherwise healthy individuals susceptible to COVID-19 (Cohen, 2021).

Novel coronavirus disease of 2019, often referred as COVID-19 was declared pandemic in March, 2020 (Cucinotta and Vanelli, 2020). Since the virus was reported, there are 119,220,681confirmed cases of COVID-19, including 2,642,826 deaths worldwide as of 5:13 pm CET, 14 March 2021 (WHO, 2020b). COVID-19 is caused by SARS-CoV-2 (novel coronavirus), which has 82% genome similarity with Severe Acute Respiratory Syndrome (SARS) causing virus, SARS-CoV (Chan et al., 2020). SARS-CoV-2 spreads rapidly than SARS-CoV and replicates actively in upper respiratory tract (Hou et al., 2020; Sungnak et al., 2020; Wölfel et al., 2020; Ziegler et al., 2020).

Pneumocytes in lungs are invaded by SARS-CoV-2 leading to collapse of the air sacs (Zhao et al., 2010). An immune response is induced as macrophages and neutrophils rush to the site, initiating inflammation that lead to pneumonia (Wang et al., 2020; Zeng et al., 2020). Death in severe cases occurs because of pneumonia and acute respiratory distress syndrome, where deposition in alveoli and small blood vessels around alveoli is formed impairing the gaseous exchange (Welty-Wolf et al., 2002; Levi et al., 2003; Moore et al., 2020; Tian et al., 2020). Hence, managing the immune response could be one of the treatment strategies for COVID-19.

Although with low mortality rates, there is accelerating rise in number of deaths because of rapid transmission and various mutated strains of SARS-CoV-2 (Fang et al., 2021; Leung et al., 2021; Ozono et al., 2021), resulting in stress among people. The treatments that are or were practiced for COVID-19 include Hydroxychloroquine, Remdesivir, Lopinavir-Ritonavir combination with or without interferon, Dexamethasone and convalescent plasma therapy (Antinori et al., 2020; Grein et al., 2020; Hung et al., 2020; Okour et al., 2020; Valk et al., 2020). Remdesivir is the only United States Food and Drug Administration (FDA) approved drug, which can be used in combination with Baricitinib for faster recovery (Kalil et al., 2020).

COVID-19 is a public health emergency; hence, FDA has issued Emergency Use Authorization (EUA) to a few vaccines, which are under trial. In December 2020, two vaccines, i.e., Pfizer-BioNTech COVID-19 Vaccine and Moderna COVID-19 Vaccine were granted EUA from FDA. Pfizer-BioNTech COVID-19 Vaccine was the first to receive EUA from WHO followed by Astra Zeneca/Oxford COVID-19 vaccine, manufactured by the Serum Institute of India and SKBio (WHO, 2020a). Two prominent vaccines namely, Astra Zeneca/Oxford COVID-19 under the local name of Covishield and Covaxin, developed by Bharat Biotech, India are being administered in India.

Precautionary measures such as N-95 or regular masks, hand-washing, use of sanitizers and social distancing can be effectively supplemented with vaccination and immune enhancing measures such as a good diet, physical exercises, yogic breathing exercises, and meditative techniques including mindfulness. We hypothesize that Yoga, especially the breathing technique, maybe instrumental in COVID-19 management as an adjunct approach. In this article we have discussed the psychological and physiological improvements achieved by practicing Yoga, including *Pranayama*, or meditation or both and tried to emphasize its role in COVID-19 management.

## Yoga and Breathing Techniques in Reducing the Disease Burden

### Yogic Breathing Techniques and Their Effects on Human Systems

Yoga originated in ancient India and includes physical, mental, and spiritual practices with an explicit emphasis on different breathing patterns. Yogic breathing in a controlled manner known as *Pranayama* is one of the eight limbs of traditional yoga. Furthermore, *Pranayamas* themselves are of eight types, (1) *Surya Bhedana* or Sun-piercing Breath or Right Nostril Breathing, (2) *Ujjayi* or Victorious or Ocean Breath, (3) *Sheetkari* or Hissing Breath, (4) *Sheetali* or Cooling Breath, (5) *Bhastrika* or Bellows Breath, (6) *Bhramari* or Humming Bee Breath, (7) *Moorchha* or Swooning Breath, and (8) *Plavini* or Floating Breath (Muktibodhananda, 2012). According to “*Hatha Yoga Pradipika*,” these *Pranayamas* have various benefits on the mind and body as a whole (Muktibodhananda, 2012).

*Surya Bhedana Pranayama* increases heat in body; it helps body vitality, treat anxiety, depression and lack of energy. *Ujjayi Pranayama* is good for throat, cardio-respiratory, nervous and digestive systems. *Sheetkari Pranayama* calms mind and reduces negative emotions, improves immunity, memory, purifies blood and refreshes the body (Thanalakshmi et al., 2014). *Sheetali Pranayama* can be beneficial in summers as it cools down the mind and body. It also reduces bad breath, regulates blood pressure and elevates mood. *Sheetkari* and *Sheetali Pranayamas* reduce blood pressure in hypertensives (Shetty, 2017). *Bhastrika Pranayama* energizes mind and body by maximizing lung capacity. It helps in respiratory problems such as sinus, bronchitis and it also improves awareness and perceptive power of senses (Budhi et al., 2019). *Bhramari Pranayama* relieves tension, anger and anxiety, reduces blood pressure, diminishes headache and migraines, and improves concentration and memory (Kuppusamy et al., 2018). *Moorchha Pranayama* promotes happiness of mind, helps the mind to draw inward, removes body fat and reduces muscle weakness. *Plavini* is an advanced *Pranayama*, which increases the body capacity to sustain without food and water for several days; helps to detoxify the body and decreases stress.

### Yogic Breathing on Stress Levels and Immunity

Emerging studies suggest the positive role of *Pranayama* in the regulation of hypothalamic-pituitary-adrenal axis and inflammatory processes (Kiecolt-Glaser et al., 2010; Kaliman et al., 2014; Bower and Irwin, 2016). They may serve as adjunct to modern approaches if not as a new avenue for the non-pharmacological treatment regime. Regular Yoga practice improves mental health, by increasing mental calmness, reducing stress, improving physical health, breathing and sleep (Cartwright et al., 2020; Haller et al., 2020; La Torre et al., 2020; Sahni et al., 2021). Meditation and mindfulness also improve psychological well-being and reduce stress and anxiety (Kwok et al., 2019; Hilcove et al., 2020; Ofei-Dodoo et al., 2020; Sadhasivam et al., 2020). These benefits are documented in the ancient Indian texts; however, the need to establish these benefits by using modern tools has triggered many scientific studies on Yoga, meditation and breathing exercises. Table 1 shows recent trial studies, which have shown reduction in stress, anxiety or depression in different study set up.

**TABLE 1:** Stress and Anxiety reduction by Yoga and mindfulness.

Clinical trial studies	Pubmed ID	Sample size	Age, years	Disorder or disease targeted or study group type	Technique	Effect on psychological stress, anxiety, or depression
Divya et al., 2021. *Global Advances in Health and Medicine*	33623726	92	43.1 ± 11.1	Health care workers	Sudarshan Kriya Yoga, workshop of 4 days followed by 40 days of self-practice	Stress, Anxiety and depression were reduced.
Sadhasivam et al., 2020. *Evidence-Based Complementary and Alternative Medicine*	32595741	348	–	Healthy participants	Bhava Spandana Program (Yoga and meditation retreat) for 4 days	Anxiety and depression were reduced. Quality of life improved
Haller et al., 2020. *Current Pharmaceutical Design*	33308110	57	51.3 ± 10.5	Breast cancer	Yoga and mindfulness technique for 66 h	Stress, Anxiety and depression were reduced.
Marshall et al., 2020. *International Journal of Environmental Research and Public Health*	32825677	13	20.8 ± 0.8	Physically active and healthy	Meditative (Hatha style) yoga for 30 min	Stress was reduced
Pattnaik et al., 2020. *Journal of Family Medicine and Primary Care*	33110832	200	–	Oral cancer patients	Yoga for 1 month	Stress was reduced
McDonnell et al., 2020. *Integrative Cancer Therapies*	33118443	49	Survivor = 66.5 ± 5.5 Family member = 60.2 ± 14.1	Survivors of non-small-cell lung cancer	Mindfulness-based intervention, Breathe Easier for 2 months	Stress, Anxiety and depression were reduced.
Grahn Kronhed et al., 2020. *Journal of Alternative and Complementary Medicine*	32543212	15	71.8 (median 72, range 63–82)	Osteoporotic Vertebral Fracture	Yoga and mindfulness technique, once a week for 10 weeks	Stress was reduced
Nirwan et al., 2020. *Journal of Complementary and Integrative Medicine*	32554833	–	–	Winter expedition members of Indian Scientific Antarctic Expedition	Yoga for 10 months	Improvement in stress-related blood markers
Sharma et al., 2020. *Journal of Alternative and Complementary Medicine*	32608989	Yoga, *n* = 33 Control, *n* = 33	Yoga = 53.2 ± 11.6 Control = 51.5 ± 8.2	Cardiovascular diseases	*Asana*, *Pranayama* and relaxation technique, 3 days per week for 12 weeks	Anxiety and depression were reduced. Quality of life improved
Goldstein et al., 2020. *Journal of American College Health*	32667254	37	20.7 ± 3.2	Undergraduate and graduate students	Sudarshan Kriya Yoga for 4 days	Stress was reduced
La Torre et al., 2020. *Journal of Clinical Medicine*	32272758	40	47.3 ± 10.9	Health care workers	Yoga and mindfulness technique for 4 weeks	Stress and Anxiety were reduced
Verma et al., 2020. *Journal of Education and Health Promotion*	32318598	33	52.4 ± 5.8	Healthy participants (principal)	Yoga for 105 min, twicw a day for 1 week	Stress was reduced
Ofei-Dodoo et al., 2020. *Journal of Occupational and Environmental Medicine*	32358474	43	–	Health care workers	Mindfulness-based yoga for 8 weeks	Stress and Anxiety were reduced
Cartwright et al., 2020. *Complementary Therapies in Medicine*	32444036	10	53.6 ± 13.2	Rheumatoid arthritis	Yoga for 16 weeks	Anxiety and depression were reduced. Quality of life improved
Hilcove et al., 2020. *Journal of Holistic Nursing*	32460584	Yoga, *n* = 41 Control, *n* = 39	Yoga = 42.4 (24 to 69) Control = 42.5 (24 to 64)	Health care workers	Mindfulness-based yoga for 6 weeks	Stress was reduced
Tong et al., 2020. *Journal of American college health*	31944898	Study1, *n* = 191 Study2, *n* = 143	Study1, 20.0 ± 1.4 Study2, 19.8 ± 1.4	Healthy undergraduates	Yoga A 60 min session in Study 1 and 12 weeks intervention in Study 2	Stress was reduced
Bressington et al., 2019. *Journal of Affective Disorders*	30711868	Yoga, *n* = 23 Control, *n* = 27	Yoga = 46.3 ± 12.8 Control = 49.3 ± 9.1	Depression	Laughter Yoga, 8 sessions over 4 weeks	Depression was reduced
Kwok et al., 2019. *JAMA Neurology*	30958514	Yoga, *n* = 71 Control, *n* = 67	Yoga = 63.7 ± 8.2 Control = 63.5 ± 9.3	Idiopathic Parkinson disease	Mindfulness-based yoga, 90 min for 8 weeks	Anxiety and depression were reduced
Miyoshi, 2019. *Journal of Occupational Health*	31368154	20	20 to 30	Health care workers	Yoga for 4 weeks	Stress was reduced
Bisht et al., 2019. Annals of Neurosciences	31975776	86	31.4 ± 7.3	Parents of retinoblastoma patients	Yoga based lifestyle intervention for 12 weeks	Stress was reduced

*The above list includes studies reported from 2019 till present.*

Yoga has been shown to improve quality of life, reduce fatigue and sleep disturbances in breast cancer patients (Cramer et al., 2017). Yoga protocol designed in India, specifically to manage diabetes can reduce co-morbidity of dyslipidemia in diabetic patients (Nagarathna et al., 2019b). Further, Yoga practice was also able to limit stress-related inflammation in women (Kiecolt-Glaser et al., 2010). Yoga intervention, including *Pranayama* and *Asana*, increases CD4, a marker of helper T cells, in HIV patients indicating improved immunity, which is prominently hampered in HIV (Joseph et al., 2015). Similarly, immunity improved in HIV-positive children/adolescents after Yoga intervention, indicated by elevation in CD4 and shifting of CD4/CD8 ratio in the normal range (Chandra et al., 2019). The pro-inflammatory cytokines IL-1β, IL-8, and monocyte chemotactic protein-1 (MCP-1) levels were reportedly reduced in the Yoga intervention (*Pranava Pranayama*) group (Twal et al., 2016). It is known that IL-1β induces the brain cyclooxygenase-2 levels, which in turn has a vital role in the stress and pain management (Gurung and Kanneganti, 2015). However, it is also important to consider the role of IL-1β as an anti-inflammatory target for chronic obstructive pulmonary disease (COPD; Dhimolea, 2010). On the other hand, IL-8, a neutrophil chemoattractant, is known to be elevated in the chronic pulmonary disorder patients including COPD, cystic fibrosis, acute respiratory disorder syndrome and asthma (Aggarwal et al., 2000; McGarvey et al., 2002). MCP-1 is known for its role in activation by macrophages, monocytes, lymphocytes, and airway epithelial cells (Lundien et al., 2002). Enhanced levels of MCP-1 have chemotactic activity on monocytes, enhance the T cells activity (Carr et al., 1994), and stimulate transforming growth factor-β and collagen synthesis (Gharaee-Kermani et al., 1996; Hogaboam et al., 1999). In rheumatoid arthritis, Yoga affects the psycho-neuro-immune axis by reduction in inflammatory cytokines and improvement in mind-body communicative markers and quality of life; disease activity was also reduced in yoga group (Gautam et al., 2020).

Breathing techniques and meditation have shown to decrease inflammation in Axial Spondyloarthritis (Buijze et al., 2019). Breathing techniques, especially the expiratory techniques improve lung functions, and immune response in bronchial asthma patients (Asimakos et al., 2018). *Bhastrika Pranayama*, the most popular *Pranayama*, improves lung function in healthy individuals (Kuppusamy et al., 2018). *Shambhavi Mahamudra kriya*, a 21-min *Isha* yoga meditation from the Isha foundation, India, include deep breathing and meditation reducing stress as was measured by Perceived Stress Scale (Peterson et al., 2017). Other breathing techniques that improve the pulmonary rehabilitation in cases of COPD include the diaphragmatic and pursed-lip breathing (Martarelli et al., 2011; Valenza et al., 2014). Both the techniques results in inspirational capacity, slow breathing rate, longer exhalation time, improved oxygen saturation, lung emptying, and dynamic hyperinflation reduction in the COPD cases (Casaburi et al., 1997; Kaminsky et al., 2017).

Studies have shown improved ventilatory function in individuals performing yoga or breathing exercise. Improved ventilatory function was reported from lowered respiratory rate and increase in tidal volume, forced vital capacity, increase in forced expiratory volume at the end of 1st second, maximum voluntary ventilation, peak expiratory flow rate and breath holding time (Vieira et al., 2014; Joo et al., 2015; Alaparthi et al., 2016; Csepregi et al., 2019). Learning to adopt new breathing techniques that help to improve lung capacity, volume and function may play a role in enhancing disease recovery, such as flu, common cold and COVID-19.

## *Isha Kriya*: A Simple Breathing Technique

Yoga uses the traditional wisdom of using breathing for self regulation. Isha Foundation, established by Sadhguru, a yogi, has developed and propagated the practice of simple yogic breathing practices including *Isha kriya* or long duration breathing technique, *Simha kriya* or short duration breathing technique, and *Shambhavi Mahamudra Kriya* (Table 2). A *kriya* is a yogic action or an inner technique, like controlling the breath. The most remarkable characteristic feature of these *kriyas* is that they are easy to learn and practice with simple instructions. They are free and app guided. *Isha kriya* is recommended to be done on empty stomach. Practicing *Isha kriya* calms mind and body, reduces stress, anxiety, and depression, energizes body, improves health and it is said to empower an individual in handling unpleasant situation around himself/herself. Recent interest is emerging on the efficacy and understanding the biological/physiological/psychological mechanisms of *Isha Kriya*. Although more research is required, the available literature points out that there is no side effect of *Isha kriya* (Narayanan et al., 2020); practicing it regularly, twice a day, is more fruitful for health. Interestingly, mood disturbances can be reduced in HCW by single-time *Isha kriya* practice shown in a pilot study examining the mood changes before and after practice in stressed HCW from surgical grand rounds and an anesthesia conference (Rangasamy et al., 2019). Isha followers often claim that individuals practicing *Isha kriya* are less susceptible to common cold and flu. However, it needs to be investigated.

**TABLE 2:** Simple Yogic breathing practices.

*ISHA KRIYA* OR LONGER DURATION BREATHING TECHNIQUE (LDBT) https://youtu.be/K4hCvdDn7Zc		
**Time interval:** ∼12–18 min		
**Preparation:** Crossed leg posture with straight spine, hands on thighs with palms facing upward, face slightly upward, mild focus between the eyebrows		
**3 Stages**		
Stage 1	Inhale/Exhale, ∼7–11 min	While inhaling mentally saying: I am not the body While exhaling mentally saying: I am not even the mind
Stage 2	Utter “aa” 7 times, ∼1 min	Producing the sound from the navel region with mouth wide open. Not very loud but enough to feel the vibrations produced by the sound
Stage 3	Sit silent for ∼5–6 min	Face slightly upward with mild focus between the eyebrows.

***SIMHA KRIYA* OR SHORT DURATION BREATHING TECHNIQUE (SDBT) https://youtu.be/lP1Y1bk1YgU**		

**Time Interval:** 3–5 min		
**Preparation:** eyes closed, sit with cross legs		
**3 Stages**		
Stage 1	Powerful inhalation/exhalation 21 times with tongue outside, ∼1–2 min	Constrictions from the throat, No abdominal jerks
Stage 2	Tongue rolled inside by pushing it back, 21 powerful inhalation/exhalation, ∼1–2 min	
Stage 3	Sit relaxed with fullness of breath for ∼30 s – 1 min	Mouth closed, Eyes closed

*Individuals aged <6 years, >70 years or who have any kind of tumor or hemorrhage in brain should do Stage 1 and Stage 2 for 12 times only in SBDT.*

*Simha kriya*, another kriya developed by Sadhguru, is said to boost the immune system, increase lung capacity, purifies body and the mind. It can even help identify individuals with respiratory problem as they are unable to perform *Simha kriya* after 4–5 days of regular practice. However, there are yet no direct studies reported in the literature.

*Shambhavi Mahamudra Kriya* is the most studied among the three kriyas, yet it has not been adequately investigated. It is a combination of *Pranayama*, yogic postures and meditation, again not prescribed in Yogic literature. A few studies have suggested that increase in the heart rate variability, sympathetic tone and vagal afferents balance is associated with *Shambhavi Mahamudra Kriya* (Selvaraj et al., 2008; Peterson et al., 2017). Though additional studies are warranted, one of the studies provides enough evidence of the relaxation with decreased stress and increased well-being (Sinha et al., 2013; Peterson et al., 2017). Due to COVID-19 pandemic interest in such techniques has re-emerged especially with regard to the efficacy and understanding the biological/physiological mechanisms of *Isha Kriya*.

In contrast to above kriyas, *Sudarshan kriya* (SKY), evolved by Art of Living, is well studied and has shown to reduce stress and improve autonomic nervous system, immunity and well-being (Sharma et al., 2003; Brown and Gerbarg, 2005; Zope and Zope, 2013; Chandra et al., 2017; Mathersul et al., 2019). SKY can improve depression and can be beneficial for Post-traumatic stress disorder (Janakiramaiah et al., 2000; Katzman et al., 2012; Seppälä et al., 2014). SKY is beneficial in maintaining oxygen saturation in the practitioners at extreme high-altitude environment and thus reduces the risk of developing high-altitude related disorders (unpublished data). A recent study has shown reduction in stress, anxiety and depression among HCWs during COVID-19 pandemic after SKY intervention (Divya et al., 2021).

Such modifications based on yogic knowledge, require longitudinal randomized trials in comparison to established techniques for efficient integration (Nagendra et al., 2019; Nagarathna et al., 2019a). These breathing techniques are taught by different Yoga schools in India like Isha foundation, Art of Living and other such schools. The followers of these foundations constitute large numbers, either using these breathing techniques or obtaining training in the same. Therefore, it is easy to recruit a sufficient sample size and test such techniques as compared to the basic Yoga techniques. These *kriyas* often face barriers as there is an attempt for their application as a health care technique by general population (Mishra et al., 2020a, c).

Practicing *Isha Kriya* regularly, twice a day, is advocated to be fruitful for health. Isha foundation states that individuals practicing *Isha kriya* are less susceptible to common cold and flu. Hence, the combination of *Isha kriya* and *Simha kriya* are often apprised as important non-pharmacological strategy to manage COVID-19. Researchers recommend breathing techniques in COVID-19 management by boosting the immunological response, strengthening respiratory system and improving the immune response (Feng et al., 2020; Khawam et al., 2020). The combined practice might improve COVID-19 by reducing stress, improving immunity, increasing lung capacity, reducing inflammation, and improving wellbeing. However, no proven stress reduction or immune enhancement by administrating yogic breathing such as *Isha kriya* has been shown. Unlike SKY, despite reported benefits from *Isha kriya* practitioners, comprehensive controlled trials haven’t been undertaken.

We have earlier reported that pre-diabetics are more stressed than diabetic patients and a yoga intervention, i.e., Diabetes Yoga Protocol, which include breathing exercises, can slow down, if not halt, the conversion of pre-diabetic condition to diabetics by altering the stress responses (Mishra et al., 2020b). Thus, successful management of the co-morbidities may reduce the risk of COVID-19. The aforementioned simple *kriyas* are designed by Sadhguru to include effectiveness of modulated breathing in everyday routine and can thus reduce the risk of co-morbidities such as diabetes, hypertension and may be COVID-19. A doctor from United Kingdom suggested simple breathing technique, which is popular online, for getting relief from COVID-19 by encouraging gaseous exchange and oxygenation; this technique does not prevent or cure COVID-19 (Hamzelou, 2020).

As it is often argued among Yoga scholars that *Isha kriya* and *Simha kriya* may be useful for COVID-19 patients, a randomized pilot study is imperative to study the role of *Isha kriya* and *Simha kriya* with the biological/physiological therapeutic efficacy. In order to further evaluate the efficacy of *Isha kriya* and *Simha kriya* in COVID-19 a randomized case-control trial is in progress (Trial registration no. CTRI/2020/10/028195).

## Psychological Stress and Immune Response

The first report of immunosuppressive effect of psychological stress was approximately 50 years ago (Herbert and Cohen, 1993). A meta-analysis of 293 independent studies spanning 30 years has revealed that immunity is affected by acute and chronic stressors (Segerstrom and Miller, 2004). While acute stress suppresses some aspects of adaptive immunity and enhances some parameters of natural immunity, chronic stress suppresses cellular as well as humoral immunity. Brief but significant stressors, ranging from student examination to current COVID-19 pandemic, have potential to suppress the cellular immunity. Loneliness has also been shown to enhance stress and inflammation under acute stress conditions (Jaremka et al., 2013; Ozamiz-Etxebarria et al., 2020). Such observations may be seen in individuals who are quarantined, COVID-19 positive, convalescent or those suffering from loneliness (during lockdown), increasing their risk of suffering from severe COVID-19 infection.

Asthma, the respiratory and common allergic disease of 21st century, is associated with psychological stress, anxiety and sadness and has a bidirectional association with panic (Lehrer et al., 2002; Hasler et al., 2005). Negative emotions such as anger and hostile behavior have negative effect on physiology including decline in lung function (Kubzansky et al., 2006). These can be avoided by Yogic interventions.

Studies have shown that psychological stress can increase risk of diabetes, upper respiratory infection and cancer, and has role in progression of cancer (Cohen et al., 2002; Afrisham et al., 2019). Presence of chronic diseases, especially respiratory disorder together with stress may enhance the degree of COVID-19, which may be further aggravated by presence of obesity. Respiratory function is decreased in obese due to decreased lung volume and accumulation of cytokine producing adipocytes (Mafort et al., 2016). It is to be noted that stress and obesity are interrelated because stress can lead to obesity (overeating response) or vice-versa (van der Valk et al., 2018). Nevertheless, both stress and obesity have adverse effect on respiratory function, which may increase the risk and severity of COVID-19. Yogic breathing is an important tool to improve oxygen saturation (Mason et al., 2013).

Furthermore, psychological stress is associated with enhanced susceptibility for viral infections (Perez et al., 2012). Natural killer cells tend to decrease under stress (Ma et al., 2013), which provides immunity until seroconversion and availability of IgG and IgM antibodies to neutralize SARS-CoV-2 in middle or later stages of COVID-19 (Xiang et al., 2020). Apparently, wound healing is also impaired under stress as indicated by increased expression of genes related to cell cycle arrest, apoptosis, and inflammation in wound site neutrophils (Roy et al., 2005).

Age is another risk factor of COVID-19 as immunity decreases with age (Nicholson, 2016). Hence, it is evident that presence of psychological stress in HCWs, quarantined individuals and COVID-19 positive cases may considerably hamper their ability to withstand COVID-19, both mentally and physically. Thus, *Pranayama* and other breathing techniques may play a substantial role in managing stress and improve immunity that might be beneficial in managing COVID-19 response, especially when patient is obese or aged and/or has chronic respiratory or other disorders. Moreover, use of mask is also an added challenge to a large number of people who are not able to breathe; thus, such yogic breathing can help.

We hypothesize that simple and controlled breathing techniques, such as *Isha kriya* or *Simha kriya*, may have role in managing COVID-19-related stress and immune response, which might be helpful in prevention or treatment as an additional and indirect approach. However, the direct involvement of Isha kriya or Simha kriya in psychological and physiological effects needs to be verified. Of note, standard precautionary measures and available drugs and vaccines are the major contributors in COVID-19 prevention and treatment.

## Discussion

Yoga may be instrumental in managing COVID-19 related stress and regulate immunity and inflammation. However, it is important to note that the key factors to control COVID-19 are prevention of the contagion, by following standard norms such as use of N-95 or regular masks, hand-washing, use of sanitizers and social distancing. Moreover, vaccines are readily available and administered in almost all countries and trials are about to complete thereby the severity of this pandemic might reduce in coming time. However, emergence of new strains of SARS-CoV-2 is of concern, as the presently available vaccines might be unresponsive toward these strains. Yoga practices, which enhance immune system, apparently reduce inflammation and related stress (either associated with infection or loneliness during quarantine), and may aid in management of COVID-19 patients, convalescent cases and HCWs, require validation through randomized clinical trial.

**FIGURE 1:**
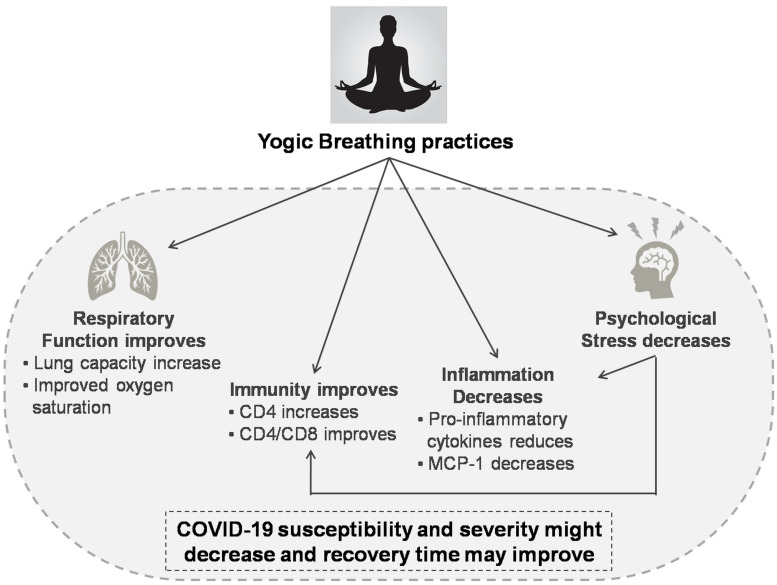
Effects of Yogic breathing and probable advantage in COVID-19. Abbreviations: CD4, cluster of differentiation 4; CD8, cluster of differentiation 8; and MCP-1, Monocyte chemoattractant protein-1.

Immunity is adversely affected by increased stress. Psychological stress is often reported among the COVID-19 positive patients and those recovering from it, quarantined individuals and HCWs (Luo et al., 2020; Ornell et al., 2020; Pfefferbaum and North, 2020; Halperin et al., 2021). Therefore, it becomes crucial not only to improve immunity but also to reduce stress. Breathing techniques are known to improve the lung functions, oxygen saturation and improved cardiovascular functions. Liuzijue exercise that includes controlled breathing was able to improve pulmonary function and quality of life in discharged COVID-19 patients (Tang et al., 2021). The best precautionary approach to cope up with COVID-19 is by enhancing immunity and lung functions. However, lack of specific Yoga modules or *kriyas* precludes extrapolation of controlled trials and their consequent translation. Several ongoing randomized control trials, including our study, aims to investigate the efficacy of various breathing techniques in COVID-19 (Lai et al., 2020; Weiner et al., 2020; Zhang et al., 2020). Figure 1 summarizes the effects of Yogic breathing techniques.

COVID19 outbreak and immediate requirement of precautionary measures and treatment has resulted in exploration of breathing techniques, including those outside the field of yogic texts. This may also act as a tool to manage COVID-19, especially in mild and moderate cases. However, drugs and vaccine are required to efficiently treat COVID-19. Such breathing techniques are easy to access during the lockdown, including the HCWs, the quarantined and COVID-19 positive patients, through digital platforms. The yoga scholars at Post Graduate Institute of Medical Education and Research, Chandigarh, India have spearheaded such a digital interface with educational programs that prompt scientific analysis of mindfulness programs via Facebook page, “Yoga Scholar PGIMER”^1^. The *Isha kriya* breathing technique was discussed by Sadhguru in one such program^2^.

Yogic breathing practice such as *Isha kriya* and *Simha kriya* are said to be simple to learn and do not require special training and supervision to execute. The combined practice only takes 15–20 min. Hence, *Isha Kriya* and *Simha kriya* must be evaluated for their efficacy through controlled trials.

## Data Availability Statement

Publicly available datasets were analyzed in this study. This data can be found here: https://www.neuroscienceresearchlab.org/.

## Author Contributions

MR wrote the first draft. BS, PA, and AA contributed to conception and design of the review. PM edited and critically reviewed the manuscript. All authors contributed to manuscript revision, read, and approved the submitted version.

## Conflict of Interest

The authors declare that the research was conducted in the absence of any commercial or financial relationships that could be construed as a potential conflict of interest.

## Footnotes

1 https://www.facebook.com/YogaScholarPGIMER/

2 https://www.facebook.com/YogaScholarPGIMER/videos/235392504240583/

## Abbreviations

ACE2: Angiotensin-converting enzyme 2
ARDS: Acute respiratory distress syndrome
CD4: Cluster of differentiation 4
CD8: Cluster of differentiation 8
COVID-19: Coronavirus Disease 2019
COX2: Cyclooxygenase 2
HCWs: Health care workers
HDAC2: Histone Deacetylase 2
HDAC3: Histone Deacetylase 3
HDAC9: Histone Deacetylase 9
LDBT: Isha kriya or long duration breathing technique
MCP-1: Monocyte chemoattractant protein-1
RIPK2: Receptor-interacting serine/threonine-protein kinase 2
SARS: Severe Acute Respiratory Syndrome
SARS-CoV-2: Severe acute respiratory syndrome coronavirus 2/novel coronavirus
SDBT: Simha kriya or short duration breathing technique.
